# Supplementary material for: How the motor system copes with aging: a quantitative meta-analysis of the effect of aging on motor function control
Source: Commun Biol. 2022 Jan 20;5:79. doi: 10.1038/s42003-022-03027-2 (PMC8776875; doi:10.1038/s42003-022-03027-2)
Supplement: Supplementary file 2 — Supplementary Information [file 42003_2022_3027_MOESM2_ESM.pdf]

Supplementary materials

How the motor system copes with aging: a quantitative meta-analysis of the effect of aging on motor function control

Laura Zapparoli[1,2], Marika Mariano[1], Eraldo Paulesu[1,2]

[1] Psychology Department and NeuroMi – Milan Centre for Neuroscience, University of Milano-Bicocca, Milan, Italy

[2] IRCCS Orthopedic Institute Galeazzi, Milan, Italy

Corresponding author

Dr Laura Zapparoli

Psychology Department

University of Milano-Bicocca

Email: laura.zapparoli@unimib.it

Supplementary Note

Performance-specific clusters

Four clusters, located in the right cerebellum (CL42), vermis (CL43), pallidum (CL47) and thalamus (CL57), were associated with an equal performance of the two groups. On the other hand, the left postcentral gyrus (CL27), the right cerebellum (CL49), and right postcentral gyrus (CL65) were specifically associated with a lower performance in the elderly group (see Table 2 and Figure S1). A further classification of peaks forming the sensorimotor clusters based on the effector/body side is available in Table S3.

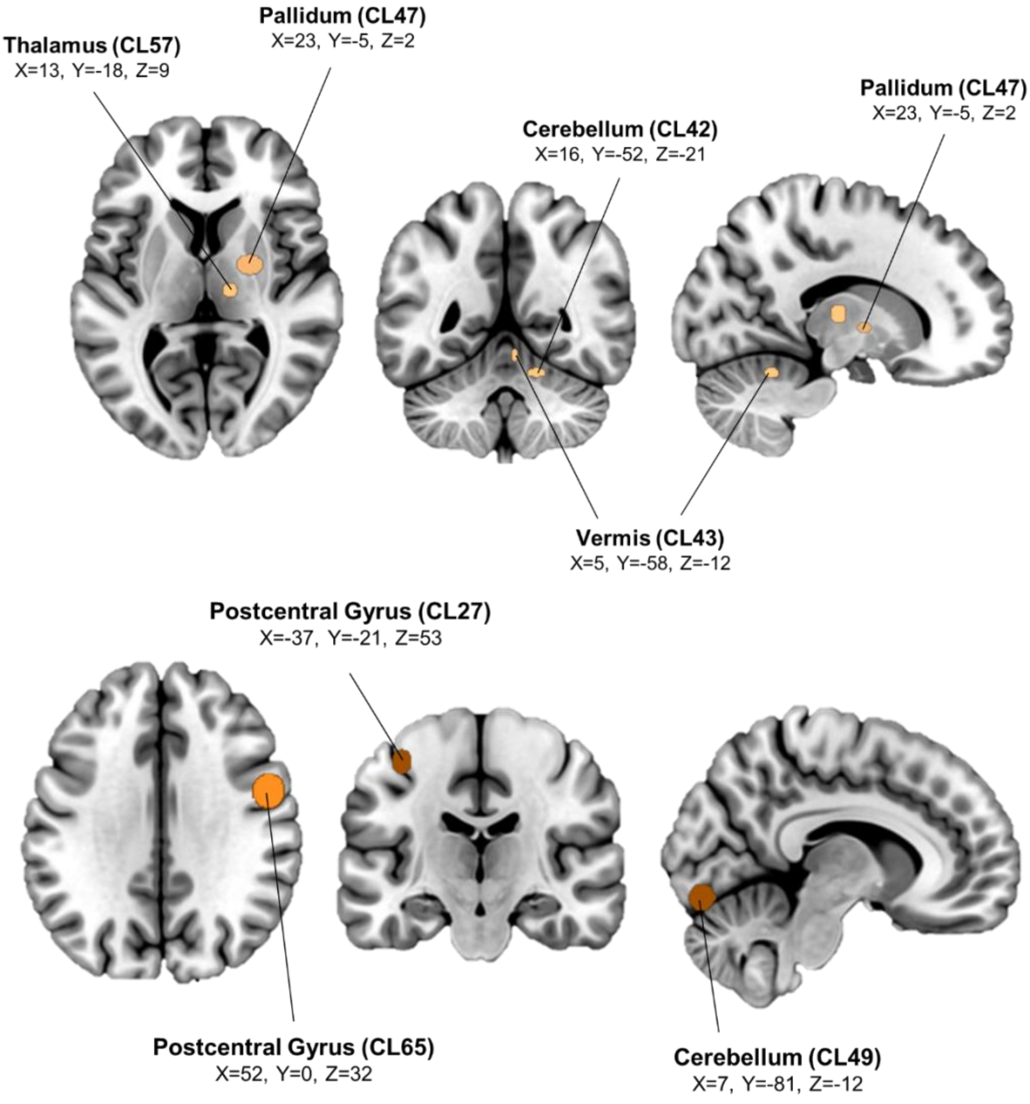

Supplementary Figure 1. Distribution of clusters showing a significant main effect of performance (upper figure: equal performance; lower figure: declined performance of elderly subjects).

Task-specific clusters

There were six clusters significantly associated with motor execution tasks: these were in the left cerebellum (CL4), supramarginal gyrus (CL11), postcentral gyrus (CL27 and CL29) and in the right postcentral gyrus (CL45) and pallidum (CL47). On the other hand, eight clusters were associated with cognitive motor tasks (e.g., motor imagery, motor observation and motor prediction): these were in left calcarine fissure (CL6), the left precentral gyrus (CL28), the left inferior parietal lobule (CL30), the right cerebellum (CL49), the right precentral gyrus (CL51), the right superior temporal gyrus (CL60 and CL61), and the right inferior temporal gyrus (CL66, see Table 2 and Figure S2). A further classification of peaks forming the sensorimotor clusters based on the effector/body side is available in Table S3.

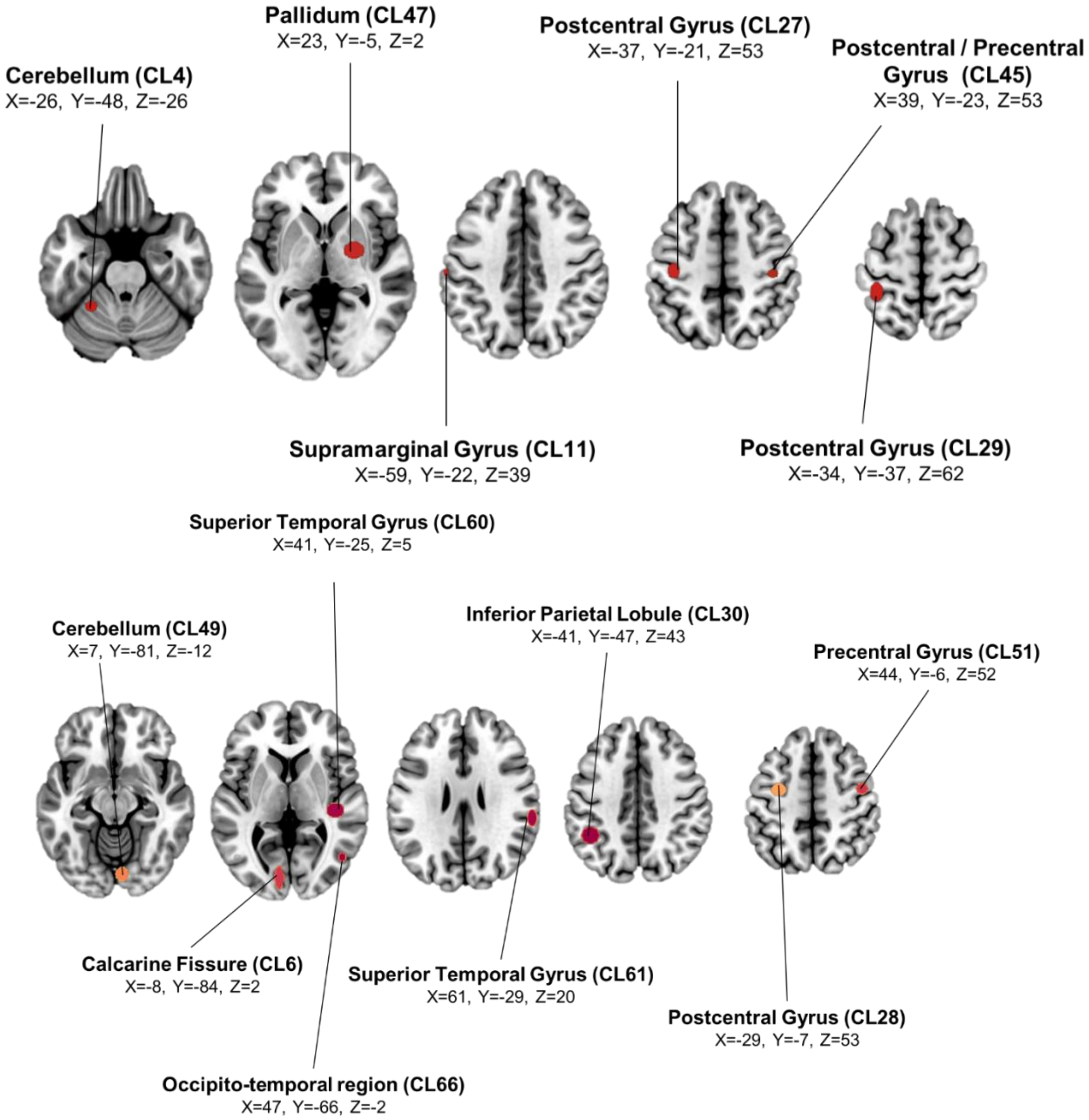

Supplementary Figure 2. Distribution of clusters showing a significant main effect of task (upper figure: motor execution tasks; lower figure: cognitive motor tasks).

Performance-by-Task interaction

We found four clusters characterized by a significant performance-by-task interaction effect. These were in the left calcarine fissure (CL6), in the right occipito-temporal region (CL66), in the right precentral/postcentral gyrus (CL45) and in the left precentral/postcentral gyrus (CL12, Table 2, Figure S4). The interaction plots show that the left calcarine fissure (CL6) and the right occipito-temporal region (CL66) are more frequently activated when the performance between elderly and young is non-equal, but only for explicit motor execution tasks. Vice versa, in case of cognitive motor tasks, this cluster is more active when the performance of Elderly and Young people is equal. An opposite pattern characterized the left precentral/postcentral gyrus (CL12): this cluster is more active when the performance is equal considering tasks requiring explicit motor execution and it is more active when the performance is not equal in case of cognitive motor tasks. Differently, the right precentral/postcentral gyrus (CL45) is more activated when the performance of the two groups is equal in both motor execution and cognitive motor tasks (see Table 2 and Figure S3). A further classification of peaks forming the sensorimotor clusters based on the effector/body side is available in Table S3.

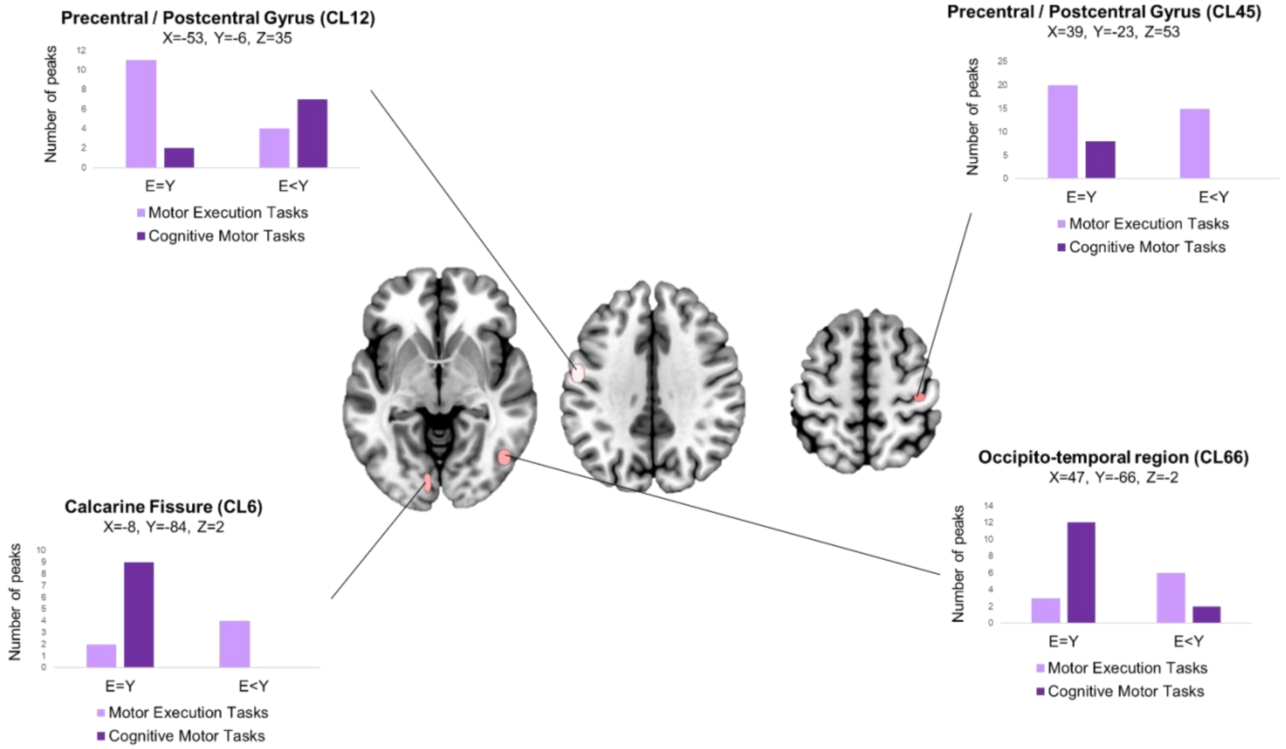

Supplementary Figure 3. Distribution of clusters showing a significant Task by Performance interaction effect. Plots source data are provided as a Supplementary File.

Supplementary Methods

The flowchart of the studies selection is shown in Figure S4.

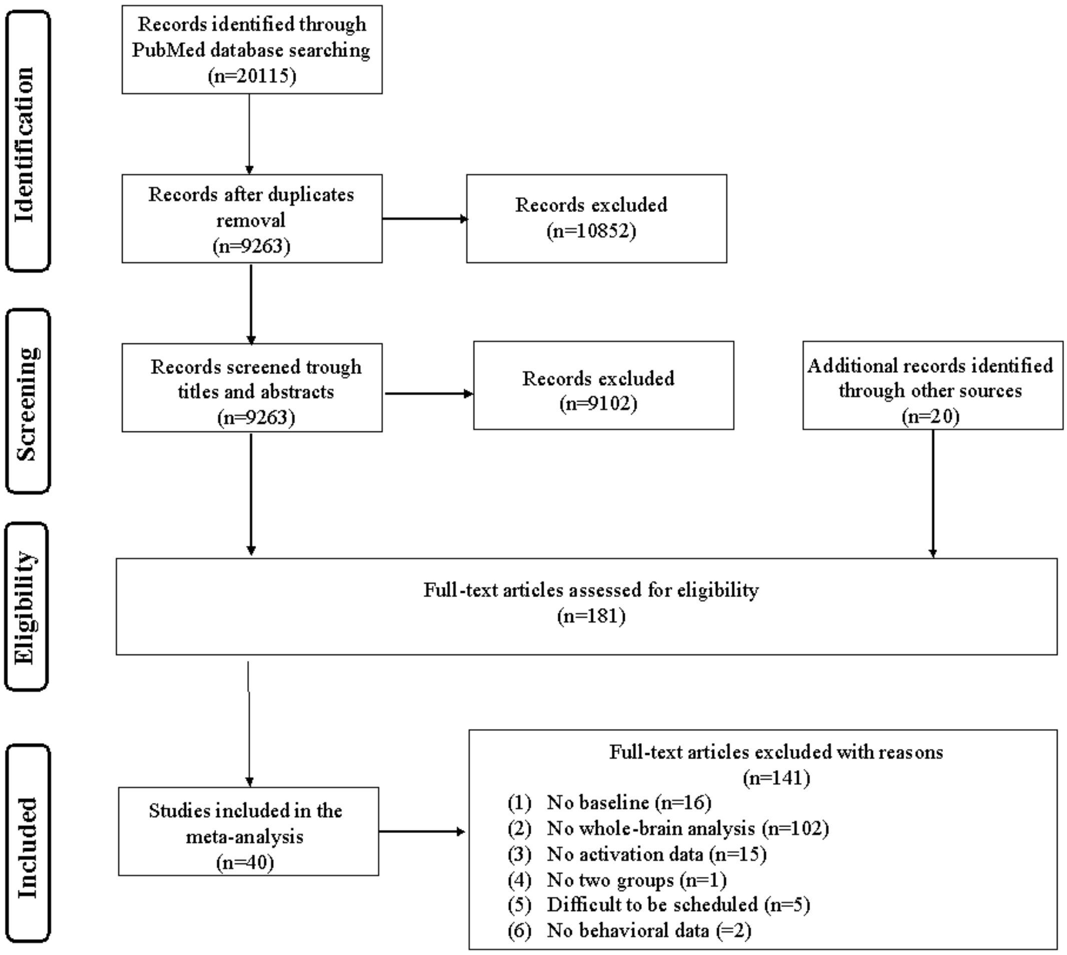

Supplementary Figure 4. Flow chart of study selection.
