# Supplementary material for: How the motor system copes with aging: a quantitative meta-analysis of the effect of aging on motor function control
Source: Commun Biol. 2022 Jan 20;5:79. doi: 10.1038/s42003-022-03027-2 (PMC8776875; doi:10.1038/s42003-022-03027-2)
Supplement: Supplementary file 3 — Description of Additional Supplementary Files [file 42003_2022_3027_MOESM3_ESM.pdf]

Description of Additional Supplementary Files

File name: Supplementary Data 1

Description: Neuroimaging studies included in the current meta-analysis. For each study, we report the first author, the publication's year, the technique used, the type of task, the performance of the two groups and the details of the experimental paradigm.

File name: Supplementary Data 2

Description: Details of the peaks forming clusters showing a significant Group Effect, Group-by-Performance and Group-by-Task effects.

File name: Supplementary Data 3

Description: Details of the peaks forming M1/S1 clusters showing a significant Group Effect, Group-by-Performance and Group-by-Task effects.

File name: Supplementary Data 4

Description: Source data for plots in Figures 2 – 4 and Supplementary Figure 3.
