# Supplementary material for: How the motor system copes with aging: a quantitative meta-analysis of the effect of aging on motor function control
Source: Commun Biol. 2022 Jan 20;5:79. doi: 10.1038/s42003-022-03027-2 (PMC8776875; doi:10.1038/s42003-022-03027-2)
Supplement: Supplementary file 8 — Reporting Summary [file 42003_2022_3027_MOESM8_ESM.pdf]

# nature portfolio

Corresponding author(s): Dr Laura Zapparoli

Last updated by author(s): Dec 9, 2021

# Reporting Summary

Nature Portfolio wishes to improve the reproducibility of the work that we publish. This form provides structure for consistency and transparency in reporting. For further information on Nature Portfolio policies, see our Editorial Policies and the Editorial Policy Checklist.

## Statistics

For all statistical analyses, confirm that the following items are present in the figure legend, table legend, main text, or Methods section.

| n/a | Confirmed | |
|---|---|---|
| ☐ | ☒ | The exact sample size (*n*) for each experimental group/condition, given as a discrete number and unit of measurement |
| ☐ | ☒ | A statement on whether measurements were taken from distinct samples or whether the same sample was measured repeatedly |
| ☐ | ☒ | The statistical test(s) used AND whether they are one- or two-sided<br>*Only common tests should be described solely by name; describe more complex techniques in the Methods section.* |
| ☒ | ☐ | A description of all covariates tested |
| ☒ | ☐ | A description of any assumptions or corrections, such as tests of normality and adjustment for multiple comparisons |
| ☐ | ☒ | A full description of the statistical parameters including central tendency (e.g. means) or other basic estimates (e.g. regression coefficient) AND variation (e.g. standard deviation) or associated estimates of uncertainty (e.g. confidence intervals) |
| ☐ | ☒ | For null hypothesis testing, the test statistic (e.g. $F$, $t$, $r$) with confidence intervals, effect sizes, degrees of freedom and $P$ value noted<br>*Give P values as exact values whenever suitable.* |
| ☒ | ☐ | For Bayesian analysis, information on the choice of priors and Markov chain Monte Carlo settings |
| ☒ | ☐ | For hierarchical and complex designs, identification of the appropriate level for tests and full reporting of outcomes |
| ☒ | ☐ | Estimates of effect sizes (e.g. Cohen's $d$, Pearson's $r$), indicating how they were calculated |

*Our web collection on statistics for biologists contains articles on many of the points above.*

## Software and code

Policy information about availability of computer code

| | |
|---|---|
| Data collection | No software was used for data collection (see the methods section of the paper for the details). |
| Data analysis | Data analsyes were conducted by using the following softwares: GingerALE (https://brainmap.org/ale/) and CluB (https://osf.io/4b2pc/wiki/home/). |

For manuscripts utilizing custom algorithms or software that are central to the research but not yet described in published literature, software must be made available to editors and reviewers. We strongly encourage code deposition in a community repository (e.g. GitHub). See the Nature Portfolio guidelines for submitting code & software for further information.

## Data

Policy information about availability of data

All manuscripts must include a data availability statement. This statement should provide the following information, where applicable:
- Accession codes, unique identifiers, or web links for publicly available datasets
- A description of any restrictions on data availability
- For clinical datasets or third party data, please ensure that the statement adheres to our policy

The datasets generated during and/or analysed during the current study are available from the corresponding author on reasonable request.

# Field-specific reporting

Please select the one below that is the best fit for your research. If you are not sure, read the appropriate sections before making your selection.

☐ Life sciences ☒ Behavioural & social sciences ☐ Ecological, evolutionary & environmental sciences

For a reference copy of the document with all sections, see nature.com/documents/nr-reporting-summary-flat.pdf

# Behavioural & social sciences study design

All studies must disclose on these points even when the disclosure is negative.

**Study description**

To address the effects of aging on motor control, we performed a quantitative meta-analysis of forty functional brain-imaging studies, considering not only the age factor, but also the specific nature of the motor task and the level of the behavioral performance.
Our meta-analytical approach involves a series of analytical steps starting from the identification of the raw data (data collection and data preparation), followed by hierarchical clustering and statistical inferences on the clusters, which comprise a cluster composition analysis. These procedures are described in detail in the methods section of our paper.

**Research sample**

The final dataset submitted to meta-analysis comprised 1349 participants, 616 elderly and 607 young participants. Please note that 126 participants cannot be assigned to the young/elderly group since in the original studies participants were not divided in two groups and authors performed a regression analysis on a single group using the variable age as covariate (see the methods section of our paper). The elderly group age range was 58-80, while the age range for the young group was 21-31.

**Sampling strategy**

N/A

**Data collection**

We identified neuroimaging studies exploring the neural correlates of motor control during either motor execution tasks or non-execution cognitive motor tasks (i.e., motor imagery, motor observation and motor prediction) in young and elderly individuals, using the following procedures.
First, we entered the following queries in PubMed (https://www-ncbi-nlm-nihgov.proxy.unimib.it/pubmed/): “fMRI and ageing and action”, “fMRI and aging and action”, “fMRI and older and action”, “fMRI and age and action”, “fMRI and ageing and [motor control]”, “fMRI and aging and [motor control]”, “fMRI and older and [motor control]”, “fMRI and age and [motor control]”, “fMRI and ageing and premotor”, “fMRI and aging and premotor”, “fMRI and older and premotor”, “fMRI and age and premotor”, “fMRI and ageing and motor”, “fMRI and aging and motor”, “fMRI and older and motor”, “fMRI and age and motor”, “PET and ageing and action”, “PET and aging and action”, “PET and older and action”, “PET and age and action”, “PET and ageing and [motor control]”, “PET and aging and [motor control]”, “PET and older and [motor control]”, “PET and age and [motor control]”, “PET and ageing and premotor”, “PET and aging and premotor”, “PET and older and premotor”, “PET and age and premotor”, “PET and ageing and motor”, “PET and aging and motor”, “PET and older and motor”, “PET and age and motor”, “neuroimaging and ageing and action”, “neuroimaging and aging and action”, “neuroimaging and older and action”, “neuroimaging and age and action”, “neuroimaging and ageing and [motor control]”, “neuroimaging and aging and [motor control]”, “neuroimaging and older and [motor control]”, “neuroimaging and age and [motor control]”, “neuroimaging and ageing and premotor”, “neuroimaging and aging and premotor”, “neuroimaging and older and premotor”, “neuroimaging and age and premotor”, “neuroimaging and ageing and motor”, “neuroimaging and aging and motor”, “neuroimaging and older and motor”, “neuroimaging and age and motor”. The initial set of studies included 20115 papers, updated to February 2021.
Second, after the removal of duplicates, we ran a preliminary selection based on the titles and abstracts of the papers, through which we included the studies that matched the following criteria:
● Studies including both healthy young and older adults.
● Studies reporting whole-brain activation peaks (no region-of-interest analyses) either from each group independently or from contrasts of the two groups.
● Data reported using stereotactic coordinates (either MNI or Talairach atlases).
● Task-based fMRI studies (no resting-state studies).
● Univariate statistical analyses.

Conversely, we excluded the studies that matched the following criteria:
● Studies that used neuroimaging methods other than task-based PET/fMRI studies, such as resting-state fMRI, PET, SPECT, or other non-fMRI procedures, to exclude variability across different neuroimaging findings.
● Studies that assessed the effect of medication or other treatments without reporting fMRI data at baseline.
● Studies analyzed with a priori region of interest approach.

**Timing**

The initial set of studies included 20115 papers, updated to February 2021.

**Data exclusions**

We excluded the studies that matched the following criteria:
● Studies that used neuroimaging methods other than task-based PET/fMRI studies, such as resting-state fMRI, PET, SPECT, or other non-fMRI procedures, to exclude variability across different neuroimaging findings.
● Studies that assessed the effect of medication or other treatments without reporting fMRI data at baseline.
● Studies analyzed with a priori region of interest approach.
See the flowchart illustrated in Figure S4.

| | |
|---|---|
| Non-participation | N/A |
| Randomization | N/A |

# Reporting for specific materials, systems and methods

We require information from authors about some types of materials, experimental systems and methods used in many studies. Here, indicate whether each material, system or method listed is relevant to your study. If you are not sure if a list item applies to your research, read the appropriate section before selecting a response.

## Materials & experimental systems

| n/a | Involved in the study |
|---|---|
| ☒ | ☐ Antibodies |
| ☒ | ☐ Eukaryotic cell lines |
| ☒ | ☐ Palaeontology and archaeology |
| ☒ | ☐ Animals and other organisms |
| ☒ | ☐ Human research participants |
| ☒ | ☐ Clinical data |
| ☒ | ☐ Dual use research of concern |

## Methods

| n/a | Involved in the study |
|---|---|
| ☒ | ☐ ChIP-seq |
| ☒ | ☐ Flow cytometry |
| ☒ | ☐ MRI-based neuroimaging |
